# High concentrations of pharmaceuticals emerging as a threat to Himalayan water sustainability

**DOI:** 10.1007/s11356-021-18302-8

**Published:** 2022-01-08

**Authors:** Duncan J. Quincey, Paul Kay, John Wilkinson, Laura J. Carter, Lee E. Brown

**Affiliations:** 1 School of Geography & Water@Leeds, University of Leeds, Leeds, LS2 9JT UK; 2 Environment and Geography Department, University of York, Heslington, YO10 5NG York UK

**Keywords:** Water pollution, Sanitation, Emerging contaminants, Antimicrobial resistance, Antibiotics

## Abstract

The sixth UN Sustainable Development Goal, Clean Water and Sanitation, directly underpins other goals of Health, Life in Water and Sustainable Cities. We highlight that poor sanitation, exemplified through some of the highest concentrations of pharmaceuticals ever detected in rivers, will amplify societal and environmental stress where climate-induced reductions in flow are predicted. Rapidly growing urban centres with inadequate water treatment works will need to prioritise water quality improvement before supply reductions become a reality. For 23 river locations within Kathmandu City and the Annapurna region, Nepal, we show the presence of 28 of 35 monitored human-use pharmaceuticals. Concentrations of antibiotics measured in this sampling campaign in both Kathmandu City (sulfamethazine, metronidazole and ciprofloxacin) and rural locations (ciprofloxacin) are in excess of predicted no effect concentrations, suggesting these sites are at risk of proliferating antimicrobial resistance as well as affecting other ecotoxicological endpoints. It is anticipated that climate-induced reductions in flow combined with contaminated river systems will amplify future societal and environmental stress.

**Supplementary Information:**

The online version contains supplementary material available at 10.1007/s11356-021-18302-8.

## Himalayan water sustainability

The Himalaya, and in particular the Brahmaputra, Ganges and Indus basins, depends on glacier ice and snow melt for water supply, food security and energy production more than any other region in the world (Bolch et al. 2012). However, for the upper parts of the Brahmaputra, peak water (the point after which runoff will steadily decline) has already passed (Huss and Hock 2018), and reduced flows in the Ganges and Indus will follow by 2070, meaning periods of water stress are likely to become more frequent and more severe (Gain and Wada 2014). The quality of the remaining river flow will thus become critical to the long-term sustainability of the region, where an estimated 800 million people depend in some way on mountain snow and glacier-melt (Bolch et al. 2012). In most cities in the developing world, water quality is blighted by inadequate treatment (UN WATER 2015) of human and industrial waste, yet remains largely absent from the research agenda in these areas (Bai et al. 2018).

As the fastest urbanising country in South Asia (Muzzini and Aparicio 2013), Nepal relies almost exclusively on the Bagmati River and its tributaries to sustain its growing population but, despite targeted clean-up efforts in recent years, pollutants (e.g. ammonia, phosphorus) remain at critically high concentrations (Bhandari et al. 2017). Sewage waste within the city of Kathmandu is rarely treated before being discharged (Harada and Karn 2001), and most commercial and industrial units also discharge wastewater either directly into watercourses or via stormwater drains. In rural locations, contaminant loads are often lower due to reduced chemical activity; however, the deterioration of surface water quality parameters, including biological oxygen demand, dissolved oxygen, and total coliforms, have been reported in response to increasing urbanisation (Harada and Karn 2001). Human-use pharmaceuticals are widely used in both urban and rural settings with public health services delivered by tertiary hospitals in urban areas and via district hospitals, primary healthcare centres and health posts in rural areas (Bhuvan et al. 2019; Rai et al. 2001). Due to limited sewage connectivity, in Nepal, waste is commonly stored in a pit or used as a natural fertiliser, and wastewater is often recycled or used for irrigation thereby providing a means of pharmaceuticals to enter, and become omnipresent, in the environment across rural and urban settings.

To date, monitoring campaigns have documented the widespread contamination of water bodies with pharmaceuticals, albeit largely in countries with considerable wastewater infrastructure (e.g. North America, Europe), as reviewed by Hughes et al. (2013) and aus der Beek (aus der Beek et al. 2016). The extent to which pharmaceutical contamination affects the Himalaya remains largely undetermined; however, with a lack of data from both rural and urban settings. In the specific case of Nepal, the demand for water is expected to increase in response to a growing population and changing climate. Furthermore, the Bagmati River and its tributaries are the principal sources of municipal water in the Kathmandu Valley as well as holding great religious and cultural importance for Hindus. Quantifying the extent to which pharmaceutical exposure is contributing to public health and potential ecosystem effects in such environments is therefore timely, as well as being highly relevant to UN Sustainable Development Goal targets.

### Water sample collection and analysis

**Fig. 1:**
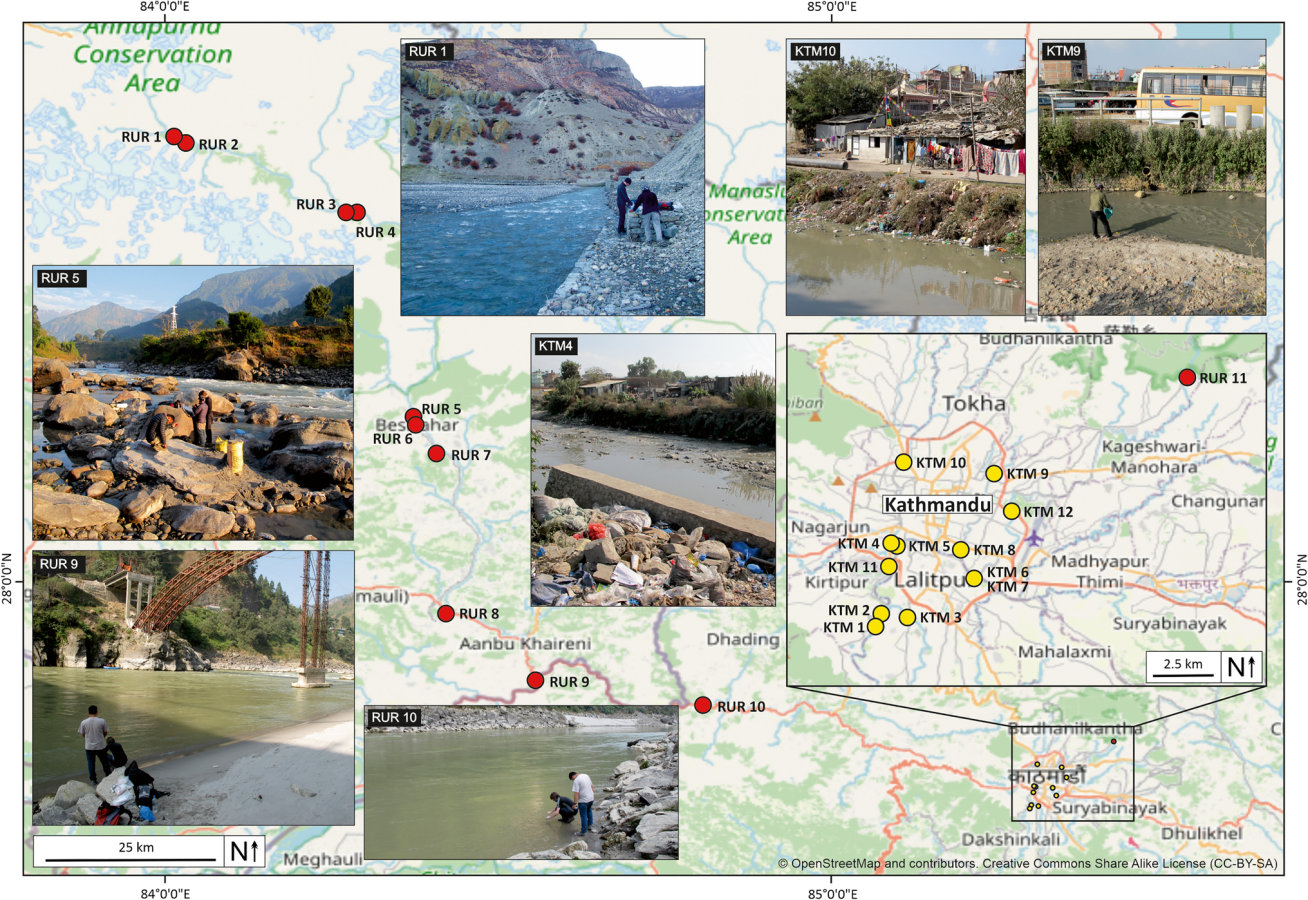
Eleven water samples were collected outside of Kathmandu Valley (classed here as ‘rural’; RUR) along a transect running from the Manang region of Annapurna (3539 m a.s.l.) to the outskirts of the city. Twelve samples were collected from within the urban area of Kathmandu City (classed here as ‘urban’ URB; ~ 1280 m a.s.l.) at major tributary junctions, around major medical institutions and at known sites of religious or social significance. Background image derived from openstreetmap.org

Water samples were collected from 11 rural sampling locations along a 160-km stretch of the Marsyangdi and Trishuli rivers, beginning in the Annapurna region and leading into Kathmandu Valley (RUR 1–11) (Fig. 1). A further 12 water samples were collected from the Bagmati and Bishnumati rivers and their tributaries within Kathmandu City (KTM 1–12) (Fig. 1). Water samples (10 mL) were aspirated directly from each sampling location using a 24-mL syringe that was rinsed 3 × with river water. A 0.7-µm GFF glass microfibre syringe filter was attached to the syringe and rinsed with 10 mL of aspirated water. The sample was discharged into 20-mL amber glass vials which were also first rinsed with filtrate. All the samples were kept in the dark at − 20° C after collection. On the day of quantitative analysis, 995 µL of each water sample was combined with 5 µL of a mixture of internal standards (each at a concentration of 80 µg/L).

Quantification of 35 selected pharmaceuticals in water samples was conducted using a high pressure liquid-chromatography/tandem mass-spectrometry (HPLC–MS/MS) method validated by Wilkinson et al. (2019). Pharmaceuticals were selected to include high usage compounds and compounds known or suspected to present ecological or human health concerns. In addition, selected compounds were also included to encompass expected high use pharmaceuticals due to regional disease pressures (e.g. antimalarials). Limits of detection ranging from 0.9 (carbamazepine) to 12.4 ng/L (gabapentin) were achieved by direct injection of 100 µL of river water (for more information see Table S1). In brief, positive electrospray ionisation was used to generate two transition ions per target pharmaceutical and internal standards; one transition for quantification and the other for confirmation. Analysis was performed on a Thermo Endura triple quardupole mass spectrometer operated in multiple reaction monitoring modes with a Phenomenex Zorbax Eclipse C18 Plus chromatography column. Mobile phase A was HPLC-grade water with 0.01 M formic acid and 0.01 M ammonium formate while mobile phase B was 100% methanol at a flow rate of 0.45 mL/min. Further instrument conditions are described in detail by Wilkinson et al. (2019). An initial chromatography column equilibration was conducted before every analytical run which comprised 20 injections of a composite sample (i.e. a combination of all samples to be analysed in a respective analytical run) to initially equilibrate the column.

Quantification occurred by a 15-point calibration using deuterated internal standards ranging from 1 to 8000 ng/L. Robust quality control measures were employed throughout sample collection and analysis. Briefly, method blanks were made with the same sample collection procedure as actual environmental samples except using ultra-pure high-performance liquid chromatography grade water. Method blanks were consistently below levels of analytical quantification. Quality controls consisting of all target analytes at concentrations of 80 and 200 ng/L were injected after every 4 samples in addition to an instrumental blank consisting of pure HPLC-grade water. Analytical tolerance was consistently within ± 15%, and instrumental blanks did not contain any detectable residue of target pharmaceuticals.

### Detection of pharmaceuticals within Kathmandu City and the Annapurna region

**Fig. 2:**
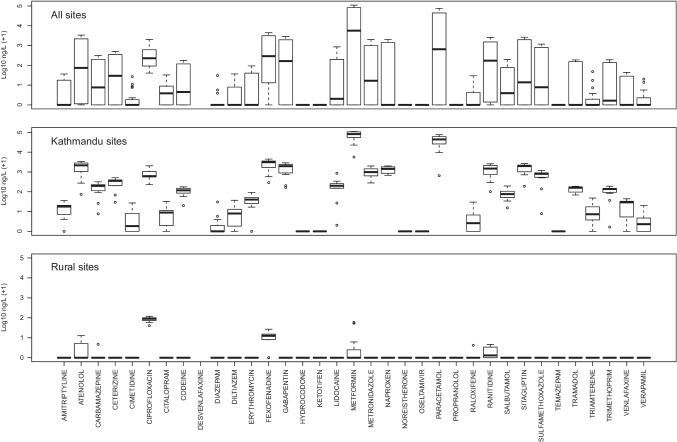
Box plots summarising log10 concentrations (ng/L) of all compounds for (a) all sites, (b) sites within Kathmandu City and (c) rural sites. Each box shows the median (horizontal bold line), interquartile range (box), 1.5* inter-quartile range (whiskers) and outliers (circles)

Across 23 river locations within Kathmandu City and the Annapurna region (Fig. 1) we show the presence of 28 human-use pharmaceuticals from a targeted list of 35 compounds. All of the 28 detected compounds were seen in the urban river samples, and 20/28 compounds were present at every urban site (Fig. 2). The highest number of compounds (27) was detected at the most downstream study location (KTM1) (Table S2). Seven pharmaceuticals were also detected in the rural sampling sites (atenolol, carbamazepine, ciprofloxacin, fexofenadine, metformin, raloxifene and ranitidine); however, in two of these cases, two compounds (carbamazepine and raloxifene) were only recorded at a single site. The highest number of compounds (six) found at a rural site were detected in a small tributary river just upstream of the town of Besisahar (RUR5) and only one compound (ciprofloxacin) was present at all 11 rural sites.

Pharmaceutical concentrations detected in the urban samples are among the highest reported in riverine studies available in the literature (Table 1). Of the 35 compounds, seven (fexofenadine, gabapentin, lidocaine, metformin, paracetamol, ranitidine and verapamil) were detected at median and maximum concentrations exceeding values reported in globally aggregated data (aus der Beek et al. 2016; Hughes et al. 2013), and of these, only verapamil was not detected at every urban site (Table 1). Concentrations of fexofenadine found in ten of our samples were two orders of magnitude higher than the mean values recorded elsewhere (Burns et al. 2018). Compounds exceeding previously published maximum values by more than one order of magnitude included metformin (eleven sites) and verapamil (three sites), while ranitidine and lidocaine each saw a three-fold increase when compared to maximum values of previous studies (at four and one site(s), respectively).

**Table 1:** Maximum recorded concentrations from previously published studies (P-Max) compared with minimum, median and maximum values from the current study

Compound	Previous studies (ng/L)		This study (KTM1 to KTM12) (ng/L)			
	P-Max (obs)	Ref	Min	Median	Max	n > P-Max
Amitriptyline	71 (35)	1	0	16.8	35.5	-
Atenolol	11,020 (1)	2	72.8	2168.2	3365.3	-
Carbamazepine	11,561 (1)	3	6.7	196.5	315.6	-
Cetirizine	530,000 (1)	4	28.5	353.4	507.6	-
Cimetidine	1338 (12)	5	0	0.9	26.0	-
Ciprofloxacin	2,500,000 (1)	4	227.2	613.0	2049.0	-
Citalopram	76,000 (1)	4	0	7.9	31.6	-
Codeine	815 (10)	6	19.2	117.0	175.6	-
Desvenlafaxine	1472 (3)^a^	7	0	0	0	-
Diazepam	140 ( −)^b^	8	0	0	29.4	-
Diltiazem	210 (8)	9	0	7.3	35.9	-
Erythromycin	4200 ( −)^a,b^	10	0	39.3	92.6	-
Fexofenadine	1144 (53)	11	288.9	**3132.2**	**4463.7**	12
Gabapentin	1887 (10)	6	162.6	**1958.2**	**2880.8**	6
Hydrocodone	910 (1)	12	0	0	0	-
Ketotifen	-^c^	-	0	0	0	-
Lidocaine	176 (7)	13	1.0	**199.3**	**855.0**	8
Metformin	3100 (5)^a^	14	**5635.4**	**82,188.0**	**110,444.9**	12
Metronidazole	7000 (1)	15	277.8	986.7	2006.1	-
Naproxen	12,300 (4)	16	675.7	1427.1	2016.3	-
Norethisterone	188 (1)	17	0	0	0	-
Oseltamivir	4600 (6)	18	0	0	0	-
Paracetamol	37,000 (18)	19	646.9	**44,967.4**	**75,979.1**	6
Propranolol	561 (5)	16	0	0	0	-
Raloxifene	7.2 (2)^d^	11	0	1.6	**28.8**	2
Ranitidine	570 (21)	20	100.6	**1479.5**	**2590.8**	10
Salbutamol	480 (8)	9	14.3	75.4	194.7	-
Sitagliptin	121(48)	11	**189.6**	**1974.9**	**2626.1**	11
Sulfamethoxazole	38,850 (1)	15	6.8	807.4	1180.1	-
Temazepam	77.8 (35)	1	0	0	0	-
Tramadol	7731 (10)	6	67.4	158.9	187.0	-
Triamterene	110 (32)	21	0	6.3	47.0	-
Trimethoprim	13,600 (23)	22	0.6	136.1	189.1	-
Venlafaxine	901 (3)^a^	7	0	28.3	43.2	-
Verapamil	0.4 (20)	23	0	**1.3**	**19.2**	8

Values are in bold emphasis where they exceed P-Max. n > P-Max denotes the number of samples within our dataset that exceed the previously published maximum values. Analysis is limited to surface water samples from rivers and streams.

1. Baker and Kaspryzk-Hordern (2013); 2. López-Serna et al. (2011); 3. Loos et al. (2008); 4. Fick et al. (2009); 5. Choi et al. (2008); 6. Kasprzyk-Hordern et al. (2008); 7. Metcalfe et al. (2010); 8. Sacher et al. (2002); 9. Barber et al. (2011); 10. Luo et al. (2011); 11. Burns et al. (2018); 12. Jones-Lepp et al. (2012); 13. Rúa-Gómez and Püttmann (2012); 14. Scheurer et al. (2012); 15. K’oreje et al. (2016); 16. Aydin and Talinli (2013); 17. Al-Odaini et al. (2010); 18. Ter Laak et al. (2010); 19. Ortiz (2010); 20. Ginebreda et al. (2010); 21. Bayerisches Landesamt für Umwelt (LfU) (2009); 22. Zhang et al. (2012); 23. Nakada et al. (2007)

^a^Mean reported value

^b^Number of observations not recorded

^c^No studies reported

^d^Below limit of quantification

Supplementary information

Inconsistent detection rates of pharmaceutical compounds in rural areas are potentially a factor of variable access to medicines across villages in the Annapurna region where factors such as distance to local health care facility and cost have been reported to impact patient access (Bhuvan et al. 2019). Antibiotics are typically self-prescribed and can be purchased over the counter (Adhikari et al. 2021);, however, limited availability of essential medicines outside of urban areas may also be a contributing factor to the lower concentrations detected in rural areas, as only 36.6% of users in health posts reported year-round availability of essential medicines in rural Nepal (Bhuvan et al. 2015). Of the compounds detected, ciprofloxacin (100% detection) remains the antibiotic of choice in rural regions of Nepal, despite concerns over the growing resistance of some organisms (Maskey et al. 2008). Similarly, detections of fexofenadine (82% detection) are unsurprising given that it is widely prescribed as an antihistamine to treat common high-altitude complaints such as dry coughs and allergies. Traces of atenolol (beta-blocker), ranitidine (H + pump inhibitor) and metformin (antihyperglycemic) detected in several rural samples are less easy to account for. Such low concentrations in rural areas could reflect prescriptions for these drugs being made in only localised populations, coupled with surface runoff from solid waste in the vicinity of the river or contamination from latrine pits sited close to the watercourse (Sah et al. 2017).

The sharp rise in contaminant concentrations recorded on the outskirts of Kathmandu is symptomatic of a wastewater infrastructure unable to cope with rapid urbanisation and high population growth rates. For the period 2014–2050, Nepal will remain amongst the top ten fastest urbanising countries in the world, with a projected annual urbanisation rate of 1.9% (Bakrania 2015), and over half of the country’s manufacturing establishments are located in the Kathmandu Valley (Harada and Karn 2001). Investment in wastewater treatment has lagged behind urban development. Major treatment plants are left unfinished, in states of disrepair, or lacking sufficient capacity to handle even household sewage waste, wastewater is often routed into the watercourses without treatment (Ashutosh et al. 2015). Recent infrastructure investments in proximity to the holy temple Pashupatinath, Kathmandu have improved wastewater treatment such that this stretch of the Bagmati River coincided with some of the lowest concentrations of pharmaceuticals recorded in our urban samples. Nevertheless, the river remains highly contaminated by untreated sewage discharges emanating from upstream (Kannel et al. 2007). Given that our most downstream sites (KTM1 and KTM2) consistently showed high numbers of detected analytes in the upper quartile (21/28 and 25/28 detections, respectively) the effect appears to be a cumulative one (Fig. 2).

Similar concerns over surface water contamination for additional chemicals are reported from major cities across the South Asian region (Sikder et al. 2013). The chronic presence of untreated sewage in rivers, as exemplified here for the Bagmati and Bishnumati Rivers with high pharmaceutical concentrations, threatens the sustainability of these rivers as the major municipal water supply, as well as pharmaceutical residues posing a possible threat to the health of those living and working on the riverbanks (Fletcher 2015).

### Wider human health and ecosystem implications

Pharmaceuticals are designed to elicit a therapeutic effect in humans and animals at low concentrations, can be taken up and accumulate in non-target species in aquatic systems (e.g. fish, bivalues) thereby presenting a potential toxicity risk (Ebele et al. 2017). A probabilistic hazard assessment of environmentally occurring pharmaceuticals predicted that 10–15% of pharmaceuticals, found in surface waters, are acutely or chronically toxic to aquatic species (Sanderson et al. 2003). Recent research has also highlighted the potential for sub-lethal toxicity whereby exposure to pharmaceuticals can affect important physiological processes involved in the growth and development of organisms. Maximum concentrations of carbamazepine and paracetamol observed at urban sampling sites were in excess of concentrations that have been previously observed to affect aquatic organisms (mussels and rotifer) through alteration of biochemical pathways, digestive lipid gland damage and increased concentration of reactive oxygen species (ROS) (Juhel et al. 2017; Martin-Diaz et al. 2009; Park et al. 2018; Solé et al. 2010) (Table S3). For example maximum reported concentrations of carbamazepine at 75 µg/L (KTM4) would exceed concentrations found to elicit oxidative stress through the generation of ROS in the rofiter, *Brachionus rotundiformis* (10 µg/L) and digestive lipid gland damage the mussel (*Mytilus galloprovincialis)* reported at 23 µg/L. Risk characterisation ratios, defined as the ratio of maximum measured concentration and the reported minimum effect concentration ranged between 3.2 and 3.3 and 3.3–7.6 for carbamazepine and paracetamol, respectively.

The presence of antibiotics in the environment has also received specific attention given that these chemicals are believed to be increasing the rate of clinically relevant antimicrobial resistance (AMR) selection (Murray et al. 2021; Stanton et al. 2020). AMR is recognised by the World Health Organisation as one of the most significant threats to global health security and is estimated to claim 700,000 lives on an annual basis as a result of drug resistance to illness (O’Neill 2014). Five antibiotics were quantified in waters (ciprofloxacin, erythromycin, metronidazole, sulfamethoxazole, trimethoprim) sampled across the urban sites (KTM1-12). Antibiotics were not detected in any of the rural sampling locations with the exception of ciprofloxacin which was detected in both Kathmandu City and the Annapurna region, albeit at approximately a factor of 10 lower in the rural locations. In response to significant knowledge gaps surrounding environmental concentrations of antibiotics that might exert selection for resistant bacteria, Bengtsson‐Palme and Larsson (2016) proposed a series of predicted no effect concentrations (PNECs) for commonly used antibiotics (Table S4). These concentrations are intended to be protective of resistance promotion in the environment. Maximum concentrations measured in this sampling campaign for ciprofloxacin and metronidazole are in excess of the respective PNEC-MICs for these compounds, suggesting these sites are at risk of selecting for AMR. Specifically, ciprofloxacin was detected at all sites, including the rural locations, with the exception of RUR9, at concentrations equal to or in excess of the PNEC-MIC, with detections at the most downstream site, KTM1, > 34 times the concentration proposed to select for resistance. Comparatively, concentrations of metronidazole were in excess of the reported PNEC-MIC, 0.13 ng/L, at all urban sites (KTM) with the exception of KTM3. Similarly, concentrations of ciprofloxacin and sulfamethoxazole identified at 9/12 Kathmandu City sampling locations, respectively, were equal to or in excess (2–4 times greater) of PNEC‐Environment (PNEC‐ENV) values proposed to be protective of ecological species by global pharmaceutical companies associated with the AMR-Industry Alliance (Tell et al. 2019) (Table S4).

However, it is important to recognise that the concentrations reported in this work came from a single grab sample which provides a snap shot of chemical contamination in time (Burns et al. 2018). Pharmaceutical concentrations can change over both temporal and spatial scales, due to changes in flow, seasonal usage trends and agricultural inputs. Therefore, when considering reported concentrations in the context of environmental risk it is important to acknowledge the limitations of using these data and the fact these may be isolated pollution incidences. It is clear, more research is urgently needed to build on these initial findings through long-term monitoring studies where repeated measurements can be obtained.

Achievement of the sixth UN Sustainable Development Goal, Clean Water and Sanitation, is a prerequisite for several of the other goals (e.g. Health and Well-Being, Life Below Water, and Sustainable Cities), underlining the inadequacy of wastewater treatment infrastructures for rapidly expanding cities across Kathmandu. While recent research efforts have focused on the quantity of future water supplies (Gain and Wada 2014; Huss and Hock 2018), more work is needed in order to understand the possible impact of pharmaceutical contamination on public health, water security and biodiversity in Nepal and elsewhere through a comprehensive spatio-temporal analysis of pharmaceutical concentrations in environmental media (Vörösmarty et al. 2010). Understanding and addressing such water quality problems is of utmost importance given that they are likely to pose a serious challenge to environmental sustainability and economic prosperity, in all developing countries across the world.

### Conclusions

In summary, for 23 river locations within a previously understudied area of Kathmandu City and the Annapurna region we show the presence of human-use pharmaceuticals in collected water samples, with 20/28 compounds being present at every urban site including some of the highest concentrations ever reported (Table 1). Even though urban sites typically had the highest measured concentrations, it is important that future monitoring studies should not neglect to assess levels of pharmaceutical pollution at rural sampling locations given that compounds observed at these sites in Nepal were found at concentrations equal to or in excess of concentrations proposed to select for antimicrobial resistance. This also highlights the need for a more comprehensive monitoring study where the reliability of grab samples to assess water pollution levels can be evaluated and interpreted to fully understand potential risk.

The EU Watch List highlights the need for the robust monitoring of select chemicals, and further studies on pharmaceuticals in the environment should assess pollutant levels of identified chemicals of concern (e.g. antimicrobials — sulfamethoxazole, trimethoprim) as well as other pharmaceuticals such as venlafaxine, as identified in this study. Future work should also take advantage of recent improvements in analytical tools to enable researchers to detect an increasing amount of micropollutants at even lower concentrations, including the quantification of previously understudied transformation products.

Our findings highlight the presence of pharmaceuticals in the previously understudied location of Nepal. It is anticipated that a reduction in long term water flow, as a consequence of climatic change, will mean sewage is discharged into smaller volumes of water thereby amplifying the concentrations currently seen in the environment. More work is needed in order to understand the possible impact of pharmaceutical contamination on public health, water security and biodiversity in Nepal and elsewhere using more comprehensive spatio-temporal sampling campaigns, which can show long-term trends accounting for factors such as seasonal variability pharmaceutical concentrations.

## Supplementary Information

Below is the link to the electronic supplementary material.

Supplementary file1 (DOCX 73 KB)

## Data Availability

The datasets used and/or analysed during the current study are available from the corresponding author on reasonable request.
