# Supplementary material for: High concentrations of pharmaceuticals emerging as a threat to Himalayan water sustainability
Source: Environ Sci Pollut Res Int. 2022 Jan 8;29(11):16749–57. doi: 10.1007/s11356-021-18302-8 (PMC8827227; doi:10.1007/s11356-021-18302-8)
Supplement: Supplementary file 1 — Supplementary file1 (DOCX 73 KB) [file 11356_2021_18302_MOESM1_ESM.docx]

**Supplementary Information**

**High concentrations of pharmaceuticals emerging as a threat to Himalayan water sustainability**

Duncan J. Quincey^1^, Paul Kay^1^, John Wilkinson^2^, Laura J. Carter^1*^ and Lee E. Brown^1^

^1^School of Geography & water@leeds, University of Leeds, Leeds, LS2 9JT, UK

^2^Environment and Geography Department, University of York, Heslington, YO10 5NG, UK

*corresponding author; [l.j.carter@leeds.ac.uk](mailto:l.j.carter@leeds.ac.uk); +44 (0) 113 34 33205

**Table S1.** Limits of detection and quantification achieved by direct aqueous injection high pressure liquid chromatography tandem mass spectrometry (Wilkinson et al., 2019)

| **Analyte** | **Limit of Detection (LOD)*** | **Limit of Quantification (LOQ)*** |
| --- | --- | --- |
|  | *Surface water (ng/L)* | *Surface water (ng/L)* |
| AMITRIPTYLINE | 0.95 | 1.87 |
| ATENOLOL | 3.01 | 6.02 |
| CARBAMAZEPINE | 0.7 | 1.4 |
| CETERIZINE | 2.4 | 4.88 |
| CIMETIDINE | 1.75 | 3.51 |
| CIPROFLOXACIN | 10.1 | 20.4 |
| CITALOPRAM | 0.778 | 1.56 |
| CODEINE | 1.32 | 2.64 |
| DESVENLAFAXINE | 4.33 | 8.66 |
| DIAZEPAM | 0.78 | 1.56 |
| DILTIAZEM | 0.501 | 1 |
| ERYTHROMYCIN | 1.57 | 3.13 |
| FEXOFENADINE | 4.64 | 9.29 |
| GABAPENTIN | 7.7 | 15.4 |
| HYDROCODONE | 1.65 | 3.3 |
| KETOTIFEN | 0.56 | 1.12 |
| LIDOCAINE | 1.33 | 2.66 |
| METFORMIN | 9.21 | 18.4 |
| METRONIDAZOLE | 5.79 | 11.6 |
| NAPROXEN | 17.9 | 35.8 |
| NORETHISTERONE | 6.57 | 13.1 |
| OSELTAMIVIR | 4.8 | 9.61 |
| PARACETAMOL | 21.7 | 43.4 |
| PROPRANOLOL | 7.43 | 14.9 |
| RALOXIFENE | 4.55 | 9.1 |
| RANITIDINE | 2.34 | 4.68 |
| SALBUTAMOL | 5.17 | 10.3 |
| SITAGLIPTIN | 4.17 | 8.33 |
| SULFAMETHOXAZOLE | 1.76 | 3.52 |
| TEMAZEPAM | 17.6 | 35.3 |
| TRAMADOL | 6.69 | 13.4 |
| TRIAMTERENE | 2.39 | 4.78 |
| TRIMETHOPRIM | 0.877 | 1.75 |
| VENLAFAXINE | 4.92 | 9.85 |
| VERAPAMIL | 1.19 | 2.37 |

**Table S2:** Full results from the HPLC-MS/MS analysis (see Methods). Values reported are either the detected level of the compound in ng/L or Not Detected (ND)

| Site | AMITRIPTYLINE (ng/L) | ATENOLOL (ng/L) | CARBAMAZEPINE (ng/L) | CETERIZINE (ng/L) | CIMETIDINE (ng/L) | CIPROFLOXACIN (ng/L) | CODEINE (ng/L) | DESVENLAFAXINE (ng/L) | DIAZEPAM (ng/L) | DILTIAZEM (ng/L) |
| --- | --- | --- | --- | --- | --- | --- | --- | --- | --- | --- |
| KTM 1 | 20.5 | 2808.7 | 195.6 | 473.7 | 10.0 | 2049.0 | 136.8 | ND | 4.7 | 10.0 |
| KTM 2 | 18.6 | 2823.4 | 197.5 | 420.0 | 7.6 | 1284.1 | 155.6 | ND | 2.9 | 9.5 |
| KTM 3 | 2.9 | 72.8 | 6.7 | 28.5 | ND | 545.4 | ND | ND | ND | ND |
| KTM 4 | 18.1 | 3365.3 | 238.6 | 507.6 | 6.6 | 1181.3 | 171.6 | ND | ND | 12.0 |
| KTM 5 | 8.3 | 2582.6 | 213.1 | 353.6 | 1.2 | 649.2 | 141.7 | ND | ND | 5.0 |
| KTM 6 | 6.2 | 909.6 | 91.5 | 201.5 | ND | 576.8 | 56.5 | ND | ND | 1.2 |
| KTM 7 | 6.1 | 1190.5 | 142.7 | 203.4 | ND | 290.5 | 67.6 | ND | ND | 0.5 |
| KTM 8 | 16.1 | 2987.2 | 268.8 | 353.2 | ND | 572.5 | 175.6 | ND | ND | 11.9 |
| KTM 9 | 17.5 | 2067.5 | 205.4 | 268.6 | ND | 893.2 | 117.0 | ND | ND | 4.7 |
| KTM 10 | 20.5 | 2268.9 | 134.9 | 358.3 | 1.3 | 943.3 | 111.8 | ND | ND | 14.0 |
| KTM 11 | 35.5 | 1897.5 | 315.6 | 370.0 | 26.0 | 573.1 | 105.0 | ND | 29.4 | 35.9 |
| KTM 12 | ND | 271.3 | 24.9 | 67.1 | 0.5 | 227.2 | 19.2 | ND | ND | ND |
| RUR 1 | ND | ND | ND | ND | ND | 66.0 | ND | ND | ND | ND |
| RUR 2 | ND | 12.0 | ND | ND | ND | 80.0 | ND | ND | ND | ND |
| RUR 3 | ND | 5.3 | ND | ND | ND | 101.6 | ND | ND | ND | ND |
| RUR 4 | ND | 4.7 | ND | ND | ND | 78.8 | ND | ND | ND | ND |
| RUR 5 | ND | ND | ND | ND | ND | 112.2 | ND | ND | ND | ND |
| RUR 6 | ND | 0.3 | 3.7 | ND | ND | 97.3 | ND | ND | ND | ND |
| RUR 7 | ND | 3.6 | ND | ND | ND | 57.3 | ND | ND | ND | ND |
| RUR 8 | ND | ND | ND | ND | ND | 93.3 | ND | ND | ND | ND |
| RUR 9 | ND | ND | ND | ND | ND | 39.6 | ND | ND | ND | ND |
| RUR 10 | ND | ND | ND | ND | ND | 88.8 | ND | ND | ND | ND |
| RUR 11 | ND | ND | ND | ND | ND | 118.3 | ND | ND | ND | ND |
| Site | ERYTHROMYCIN (ng/L) | FEXOFENADINE (ng/L) | GABAPENTIN (ng/L) | HYDROCODONE (ng/L) | KETOTIFEN (ng/L) | LIDOCAINE (ng/L) | METRONIDAZOLE (ng/L) | NAPROXEN (ng/L) | NOREISTHERONE (ng/L) | OSELTAMIVIR (ng/L) |
| KTM 1 | ND | 3099.4 | 2582.9 | ND | ND | 195.8 | 986.7 | 1809.3 | ND | ND |
| KTM 2 | ND | 3219.8 | 2468.4 | ND | ND | 202.8 | 1084.7 | 2016.3 | ND | ND |
| KTM 3 | 51.2 | 288.9 | 162.6 | ND | ND | 1.0 | ND | ND | ND | ND |
| KTM 4 | ND | 4463.7 | 2880.8 | ND | ND | 234.3 | 1919.4 | 1949.7 | ND | ND |
| KTM 5 | 92.6 | 3025.0 | 2220.6 | ND | ND | 228.0 | 910.9 | 1560.0 | ND | ND |
| KTM 6 | 42.6 | 1595.7 | 754.9 | ND | ND | 176.4 | 751.2 | 760.9 | ND | ND |
| KTM 7 | 40.7 | 1782.1 | 1043.8 | ND | ND | 150.1 | 409.6 | 675.7 | ND | ND |
| KTM 8 | 15.7 | 3875.9 | 2262.9 | ND | ND | 342.1 | 1324.9 | 1396.5 | ND | ND |
| KTM 9 | 24.5 | 3350.4 | 1104.8 | ND | ND | 150.1 | 1554.7 | 852.7 | ND | ND |
| KTM 10 | 37.9 | 3805.7 | 2114.1 | ND | ND | 277.1 | 2006.1 | 1457.7 | ND | ND |
| KTM 11 | 24.6 | 3165.1 | 1802.3 | ND | ND | 855.0 | 535.0 | 903.4 | ND | ND |
| KTM 12 | 51.3 | 575.2 | 201.0 | ND | ND | 25.8 | 277.8 | ND | ND | ND |
| RUR 1 | ND | 12.7 | ND | ND | ND | ND | ND | ND | ND | ND |
| RUR 2 | ND | 8.0 | ND | ND | ND | ND | ND | ND | ND | ND |
| RUR 3 | ND | 11.5 | ND | ND | ND | ND | ND | ND | ND | ND |
| RUR 4 | ND | 22.3 | ND | ND | ND | ND | ND | ND | ND | ND |
| RUR 5 | ND | 14.0 | ND | ND | ND | ND | ND | ND | ND | ND |
| RUR 6 | ND | 25.9 | ND | ND | ND | ND | ND | ND | ND | ND |
| RUR 7 | ND | ND | ND | ND | ND | ND | ND | ND | ND | ND |
| RUR 8 | ND | 7.2 | ND | ND | ND | ND | ND | ND | ND | ND |
| RUR 9 | ND | ND | ND | ND | ND | ND | ND | ND | ND | ND |
| RUR 10 | ND | 14.5 | ND | ND | ND | ND | ND | ND | ND | ND |
| RUR 11 | ND | 7.0 | ND | ND | ND | ND | ND | ND | ND | ND |
| Site | PARACETAMOL (ng/L) | PROPRANOLOL (ng/L) | RALOXIFENE (ng/L) | RANITIDINE (ng/L) | SALBUTAMOL (ng/L) | SITAGLIPTIN (ng/L) | TRAMADOL (ng/L) | TRIAMTERENE (ng/L) | TRIMETHOPRIM (ng/L) | VENLAFAXINE (ng/L) |
| KTM 1 | 63750.9 | ND | 28.8 | 1438.0 | 93.6 | 2076.1 | 174.3 | 16.3 | 138.5 | 27.4 |
| KTM 2 | 62736.8 | ND | 21.9 | 1520.9 | 112.6 | 2208.3 | 158.9 | ND | 145.9 | 43.2 |
| KTM 3 | 646.9 | ND | 4.7 | 100.6 | ND | ND | ND | 6.3 | 0.6 | ND |
| KTM 4 | 75979.1 | ND | 5.8 | 2590.8 | 122.6 | 2626.1 | 175.4 | ND | 189.1 | 29.9 |
| KTM 5 | 59728.1 | ND | 5.5 | 1532.2 | 97.8 | 1974.9 | 148.9 | ND | 140.3 | 28.3 |
| KTM 6 | 22079.6 | ND | 1.1 | 1151.2 | 32.8 | 718.4 | 67.4 | ND | 82.6 | ND |
| KTM 7 | 30659.2 | ND | 2.1 | 607.8 | 56.9 | 801.1 | 84.7 | ND | 103.2 | ND |
| KTM 8 | 53219.7 | ND | ND | 2473.8 | 75.4 | 2426.7 | 187.0 | 2.8 | 142.4 | ND |
| KTM 9 | 36715.2 | ND | ND | 1984.6 | 42.8 | 1292.8 | 95.0 | ND | 109.1 | ND |
| KTM 10 | 56021.7 | ND | ND | 2244.2 | 64.1 | 1463.5 | ND | ND | 178.9 | 33.9 |
| KTM 11 | 34448.1 | ND | ND | 926.0 | 194.7 | 2206.2 | 184.8 | 47.0 | 133.8 | ND |
| KTM 12 | 9623.0 | ND | ND | 290.7 | 14.3 | 189.6 | ND | ND | 35.6 | ND |
| RUR 1 | ND | ND | 3.2 | 2.4 | ND | ND | ND | ND | ND | ND |
| RUR 2 | ND | ND | ND | 0.2 | ND | ND | ND | ND | ND | ND |
| RUR 3 | ND | ND | ND | ND | ND | ND | ND | ND | ND | ND |
| RUR 4 | ND | ND | ND | 1.8 | ND | ND | ND | ND | ND | ND |
| RUR 5 | ND | ND | ND | ND | ND | ND | ND | ND | ND | ND |
| RUR 6 | ND | ND | ND | 3.7 | ND | ND | ND | ND | ND | ND |
| RUR 7 | ND | ND | ND | ND | ND | ND | ND | ND | ND | ND |
| RUR 8 | ND | ND | ND | ND | ND | ND | ND | ND | ND | ND |
| RUR 9 | ND | ND | ND | ND | ND | ND | ND | ND | ND | ND |
| RUR 10 | ND | ND | ND | 3.1 | ND | ND | ND | ND | ND | ND |
| RUR 11 | ND | ND | ND | 0.4 | ND | ND | ND | ND | ND | ND |

**Table S3:** Collated pharmaceutical ecotoxicity data for species in the aquatic environment. Where pharmaceuticals in this study were measured in excess of concentrations published to elicit an effect these cells have been highlighted in grey

| **PHARMACEUTICAL** | **CONCENTRATION AT WHICH EFFECT OBSERVED** | **REFERENCE** |
| --- | --- | --- |
| AMITRIPTYLINE | 0.78 - 5.55 mg/L | (Brausch et al., 2012) |
|  | 17.03 - 45.24 mg/L | (Minguez et al., 2014) |
| ATENOLOL | 38 - > 100 mg/L | (Brausch et al., 2012) |
|  | 1 - 50 mg/L | (Al-Aidaroos et al., 2017) |
| CARBAMAZEPINE | 0.01 - > 100 mg/L | (Brausch et al., 2012) |
|  | 1 - 96 ng/L | (Juhel et al., 2017) |
|  | 0.1 - 10 ug/L | (Martin-Diaz et al., 2009) |
| CETIRIZINE | NA | NA |
| CIMETIDINE | > 100 - > 1000 (740 mg/L) | (Brausch et al., 2012) |
| CIPROFLOXACIN | 10->100 mg/L | (Brausch et al., 2012) |
| CITALOPRAM | 7.5 - 32.94 mg/L | (Minguez et al., 2014) |
| CODEINE | NA | NA |
| DESVENLAFAXINE | NA | NA |
| DIAZEPAM | 4.3 - > 10,000 | (Brausch et al., 2012) |
| DILTIAZEM | 8.2 - 407 mg/L | (Brausch et al., 2012) |
| ERYTHROMYCIN | 2 - 100000 ug/L | (Lacaze et al., 2015) |
|  | 0.7 - 8.8 ug/L | Correia et al., 2018 |
| FEXOFENADINE | NA | NA |
| GABAPENTIN | > 1100 mg/L | (Brausch et al., 2012) |
| HYDROCODONE | NA | NA |
| KETOTIFEN | NA | NA |
| LIDOCAINE | NA | NA |
| METFORMIN | 64 - > 982 mg/L | (Brausch et al., 2012) |
| METRONIDAZOLE | >100 -1000 mg/L | (Brausch et al., 2012) |
| NAPROXEN | 140-690 mg/L | (Brausch et al., 2012) |
|  | 330 - 24200 mg/L | (Brausch et al., 2012) |
| NORETHISTERONE | NA | NA |
| OSELTAMIVIR | NA | NA |
| PARACETAMOL | 13-5306 mg/L | (Brausch et al., 2012) |
|  | 10-1000 ug/L | (Park et al., 2018) |
|  | 1 - 100 ug/L | (Bebianno et al., 2017) |
|  | 20 - 200 ug/L | (Solé et al., 2010) |
| PROPRANOLOL | 0.35 - 29.8 mg/L | (Brausch et al., 2012) |
|  | 100 - 1000 ug/L | (Oskarsson et al., 2014) |
|  | 1 - 1000 ug/L | (Ericson et al., 2010) |
|  | 0.3 ng/L | (Franzellitti et al., 2013) |
|  | 20 - 200 ug/L | (Solé et al., 2010) |
| RALOXIFENE | NA | NA |
| RANITIDINE | 650 mg/L | (Brausch et al., 2012) |
| SALBUTAMOL | NA | NA |
| SITAGLIPTIN | NA | NA |
| SULFAMETHOXAZOLE | 0.006 - >750 mg/L | (Brausch et al., 2012) |
|  | 0.002 - 100 mg/L | (Lacaze et al., 2015) |
|  | 60 mg/L | (Garcia et al., 2014) |
| TEMAZEPAM | NA | NA |
| TRAMADOL | 73-130 mg/L | (Brausch et al., 2012) |
| TRIAMTERENE | NA | NA |
| TRIMETHOPRIM | > 100 - 130000 mg/L | (Brausch et al., 2012) |
|  | 0.002 - 100 mg/L | (Lacaze et al., 2015) |
| VENLAFAXINE | 10E-07 -10E07 ug/L | (Estévez-Calvar et al., 2017) |
|  | 3.45 - 345 ug/L | (Fong et al., 2017) |
|  | 0.0015 - 75 mg/L | (Lacaze et al., 2015) |
| VERAPAMIL | 2.72-327 mg/L | (Brausch et al., 2012) |

**Table S4:** Collated Predicted No Effect Concentrations (PNECs) for antibiotics monitored in this study. Where antibiotics in this study were measured in excess of concentrations published to select for resistance these cells have been highlighted in grey.

|  | AMR INDUSTRY ALLIANCE (Tell et al., 2019) | AMR INDUSTRY ALLIANCE (Tell et al., 2019) | (Bengtsson-Palme & Larsson, 2016) |
| --- | --- | --- | --- |
| **PHARMACEUTICAL - ANTIBIOTIC** | PNEC-ENV (ug/L) | PNEC-MIC (ug/L) | PNEC (resistance selection) (ug/L) |
| CIPROFLOXACIN | 0.45 | 0.06 | 0.0064 |
| ERYTHROMYCIN | 0.5 | 1 | 1 |
| METRONIDAZOLE | N/A | 0.13 | 0.125 |
| SULFAMETHOXAZOLE | 0.6 | 16 | 16 |
| TRIMETHOPRIM | 100 | 0.5 | 0.5 |

**References**

Al-Aidaroos, A. M., Satheesh, S., & Devassy, R. P. (2017). Effects of pharmacological compounds on the barnacle larval development, metabolism and settlement. *International Biodeterioration and Biodegradation*, *117*, 190–196. https://doi.org/10.1016/j.ibiod.2017.01.003

Bebianno, M. J., Mello, A. C. P., Serrano, M. A. S., Flores-Nunes, F., Mattos, J. J., Zacchi, F. L., Piazza, C. E., Siebert, M. N., Piazza, R. S., Gomes, C. H. A. M., Melo, C. M. R., & Bainy, A. C. D. (2017). Transcriptional and cellular effects of paracetamol in the oyster Crassostrea gigas. *Ecotoxicology and Environmental Safety*, *144*(April), 258–267. https://doi.org/10.1016/j.ecoenv.2017.06.034

Bengtsson-Palme, J., & Larsson, D. G. J. (2016). Concentrations of antibiotics predicted to select for resistant bacteria: Proposed limits for environmental regulation. *Environment International*, *86*, 140–149. https://doi.org/10.1016/j.envint.2015.10.015

Brausch, J. M., Connors, K. A., Brooks, B. W., & Rand, G. M. (2012). Human pharmaceuticals in the aquatic environment: A review of recent toxicological studies and considerations for toxicity testing. *Reviews of Environmental Contamination and Toxicology*, *218*, 1–99. https://doi.org/10.1007/978-1-4614-3137-4_1

Ericson, H., Thorsén, G., & Kumblad, L. (2010). Physiological effects of diclofenac, ibuprofen and propranolol on Baltic Sea blue mussels. *Aquatic Toxicology*, *99*(2), 223–231. https://doi.org/10.1016/j.aquatox.2010.04.017

Estévez-Calvar, N., Canesi, L., Montagna, M., Faimali, M., Piazza, V., & Garaventa, F. (2017). Adverse effects of the SSRI antidepressant sertraline on early life stages of marine invertebrates. *Marine Environmental Research*, *128*, 88–97. https://doi.org/10.1016/j.marenvres.2016.05.021

Fong, P. P., Bury, T. B. S., Donovan, E. E., Lambert, O. J., Palmucci, J. R., & Adamczak, S. K. (2017). Exposure to SSRI-type antidepressants increases righting time in the marine snail Ilyanassa obsoleta. *Environmental Science and Pollution Research*, *24*(1), 725–731. https://doi.org/10.1007/s11356-016-7855-y

Franzellitti, S., Buratti, S., Valbonesi, P., & Fabbri, E. (2013). The mode of action (MOA) approach reveals interactive effects of environmental pharmaceuticals on Mytilus galloprovincialis. *Aquatic Toxicology*, *140*–*141*, 249–256. https://doi.org/10.1016/j.aquatox.2013.06.005

Garcia, R. N., Chung, K. W., Delorenzo, M. E., & Curran, M. C. (2014). Individual and mixture effects of caffeine and sulfamethoxazole on the daggerblade grass shrimp Palaemonetes pugio following maternal exposure. *Environmental Toxicology and Chemistry*, *33*(9), 2120–2125. https://doi.org/10.1002/etc.2669

Juhel, G., Bayen, S., Goh, C., Lee, W. K., & Kelly, B. C. (2017). Use of a suite of biomarkers to assess the effects of carbamazepine, bisphenol A, atrazine, and their mixtures on green mussels, *Perna viridis*. *Environmental Toxicology and Chemistry*, *36*(2), 429–441. https://doi.org/10.1002/etc.3556

Lacaze, E., Pédelucq, J., Fortier, M., Brousseau, P., Auffret, M., Budzinski, H., & Fournier, M. (2015). Genotoxic and immunotoxic potential effects of selected psychotropic drugs and antibiotics on blue mussel (Mytilus edulis) hemocytes. *Environmental Pollution*, *202*, 177–186. https://doi.org/10.1016/j.envpol.2015.03.025

Martin-Diaz, L., Franzellitti, S., Buratti, S., Valbonesi, P., Capuzzo, A., & Fabbri, E. (2009). Effects of environmental concentrations of the antiepilectic drug carbamazepine on biomarkers and cAMP-mediated cell signaling in the mussel Mytilus galloprovincialis. *Aquatic Toxicology*, *94*(3), 177–185. https://doi.org/10.1016/j.aquatox.2009.06.015

Minguez, L., Farcy, E., Ballandonne, C., Lepailleur, A., Serpentini, A., Lebel, J. M., Bureau, R., & Halm-Lemeille, M. P. (2014). Acute toxicity of 8 antidepressants: what are their modes of action? *Chemosphere*, *108*, 314–319. https://doi.org/10.1016/J.CHEMOSPHERE.2014.01.057

Oskarsson, H., Wiklund, A. K. E., Thorsén, G., Danielsson, G., & Kumblad, L. (2014). Community interactions modify the effects of pharmaceutical exposure: A microcosm study on responses to propranolol in Baltic Sea coastal organisms. *PLoS ONE*, *9*(4). https://doi.org/10.1371/journal.pone.0093774

Park, J. C., Yoon, D.-S., Byeon, E., Seo, J. S., Hwang, U.-K., Han, J., & Lee, J.-S. (2018). Adverse effects of two pharmaceuticals acetaminophen and oxytetracycline on life cycle parameters, oxidative stress, and defensome system in the marine rotifer Brachionus rotundiformis. *Aquatic Toxicology*, *204*, 70–79. https://doi.org/10.1016/j.aquatox.2018.08.018

Solé, M., Shaw, J. P., Frickers, P. E., Readman, J. W., & Hutchinson, T. H. (2010). Effects on feeding rate and biomarker responses of marine mussels experimentally exposed to propranolol and acetaminophen. *Analytical and Bioanalytical Chemistry*, *396*(2), 649–656. https://doi.org/10.1007/s00216-009-3182-1

Tell, J., Caldwell, D. J., Häner, A., Hellstern, J., Hoeger, B., Journel, R., Mastrocco, F., Ryan, J. J., Snape, J., Straub, J. O., & Vestel, J. (2019). Science‐based Targets for Antibiotics in Receiving Waters from Pharmaceutical Manufacturing Operations. *Integrated Environmental Assessment and Management*, *15*(3), 312–319. https://doi.org/10.1002/ieam.4141

Wilkinson, J., Boxall, A., & Kolpin, D. (2019). A Novel Method to Characterise Levels of Pharmaceutical Pollution in Large-Scale Aquatic Monitoring Campaigns. *Applied Sciences*, *9*(7), 1368. https://doi.org/10.3390/app9071368
